# Evaluating Surgical Response Times in Category 1 Major Trauma

**DOI:** 10.7759/cureus.97139

**Published:** 2025-11-18

**Authors:** Lloyed Gerard

**Affiliations:** 1 Accident and Emergency, Royal Stoke University Hospital, Stoke, GBR

**Keywords:** cadiothoracic surgery, emergency medicine, injury severity score (iss), major trauma, neurosurgery, orthopaedics and traumatology, surgical response

## Abstract

Background

Patients who activate a Category 1 (Cat1) trauma call have life-threatening injuries and require an immediate, coordinated response from a multidisciplinary trauma team. National guidelines emphasise prompt surgical review; however, in practice, surgical teams are not always alerted at the point of trauma activation. Delays may contribute to missed interventions, prolonged intensive care unit (ICU) stays, and increased mortality.

Aim

This study aims to evaluate surgical team attendance and the time interval to surgical input in Cat1 trauma cases, and to assess their association with injury severity and patient mortality. The audit also aimed to explore whether implementing a structured bleep system could improve early surgical input.

Methods

A retrospective audit was conducted at Royal Stoke University Hospital (a tertiary major trauma centre), including Cat1 trauma cases from October 2024 to April 2025. Thirty-seven patients were identified, of whom 30 (81%) met Cat1 criteria upon retrospective review. Data was extracted from electronic patient records and operative reports. Key measures included the time from the patient's arrival in the emergency department to surgical review, specialty attendance, ICU admission and length of stay, and 30-day mortality. Analyses were stratified by Abbreviated Injury Scale (AIS) scores and Injury Severity Score (ISS).

Results

Of the 30 Cat1 patients (mean age: 49.6 years, male: 77%), road traffic collisions (RTCs) were the leading mechanism of injury. The 30-day mortality rate of Cat1 trauma cases was 46.7%. Anaesthetic attendance was the highest (83%), followed by trauma and orthopaedics (T&O) (73%), cardiothoracic surgery (53%), general surgery (40%), and neurosurgery (40%). Mean arrival times varied: general surgery, 86.8 minutes; cardiothoracic surgery, 140.3 minutes; T&O, 160.4 minutes; and neurosurgery, 177.6 minutes. Damage control surgery occurred earliest in cardiothoracic cases (mean: 69 minutes) and was most delayed in T&O (mean: 231 minutes). Patients reviewed by ≤1 surgical specialty had four-fold higher mortality compared with those seen by ≥2 (66.7% versus 33.3%; odds ratio (OR): 4.0, p = 0.12). Mortality patterns differed across specialities, with delayed surgical review generally associated with poorer outcomes, although most associations did not reach statistical significance due to the limited sample size.

Conclusion

This audit highlights variable surgical response times in Cat1 trauma, with a trend towards poorer outcomes when specialist review was delayed or limited to a single team. Although most findings did not reach statistical significance, the results suggest potential clinical benefit from earlier and broader surgical involvement. Implementing a structured bleep system for immediate activation of relevant surgical specialities may improve coordination, reduce delays, and ultimately enhance outcomes in Cat1 trauma.

## Introduction

Major trauma refers to serious and/or multiple injuries that pose an immediate threat to life, risk long-term disability, or prevent a patient from returning to their baseline level of function [1]. Clinically, major trauma is often defined by an Injury Severity Score (ISS) > 15, a validated scoring system derived from the Abbreviated Injury Scale (AIS) [2]. This score often identifies patients at increased risk of morbidity and mortality, but is typically calculated later in patient care.

In the UK, major trauma care is delivered through regional trauma networks, with major trauma centres providing 24/7 access to specialist teams and facilities [3,4]. A structured trauma team is activated to rapidly assess and stabilise patients. This team encompasses a variety of specialities, such as emergency medicine, anaesthetics, surgery, radiology, and nursing [5]. For the most critically injured patients, this requires a tiered trauma team response, ensuring timely access to senior clinicians, including surgeons, within minutes of arrival [4,6]. At our institution, at Royal Stoke University Hospital, Category 1 (Cat1) trauma represents the highest level of trauma activation, reserved for the most critically injured patients requiring immediate, multi-specialty intervention. This is followed by Category 2 and Category 3 activations, which represent progressively lower levels of urgency and resource mobilisation based on the severity of the patient's injuries. Cat1 trauma frequently requires immediate consultant input across multiple specialities, damage control resuscitation, early imaging, and/or expedited transfer to emergency theatre or interventional radiology [5].

Despite national and international guidance recommending early surgical review [3,5], there is anecdotal concern that, in practice, surgical teams are not consistently alerted at the point of trauma call, particularly in Cat1 cases. Delays in surgical involvement may result in missed or postponed interventions, prolonged intensive care unit (ICU) stays, and worse patient outcomes. While the concept of the "golden hour," the first 60 minutes following traumatic injury, emphasises the critical importance of prompt medical intervention to save lives, there is currently no nationally defined threshold for the timing of surgical interventions in major trauma cases [7]. This gap in standardisation may contribute to variability in practice and potentially impact patient care.

To further investigate these concerns, this audit explores the relationship between surgical arrival times and patient outcomes across four key specialities: general surgery, trauma and orthopaedics (T&O), cardiothoracic surgery, and neurosurgery. By analysing arrival times against 30-day mortality, stratified by AIS scores, the study aims to determine whether delays in surgical involvement impact outcomes for patients who have sustained major trauma.

This audit, therefore, evaluates whether current trauma team processes meet expected standards for early surgical activation and explores whether a structured bleep system could enhance coordination, reduce delays, and ultimately improve clinical outcomes for Cat1 trauma patients.

## Materials and methods

**Figure 1:**
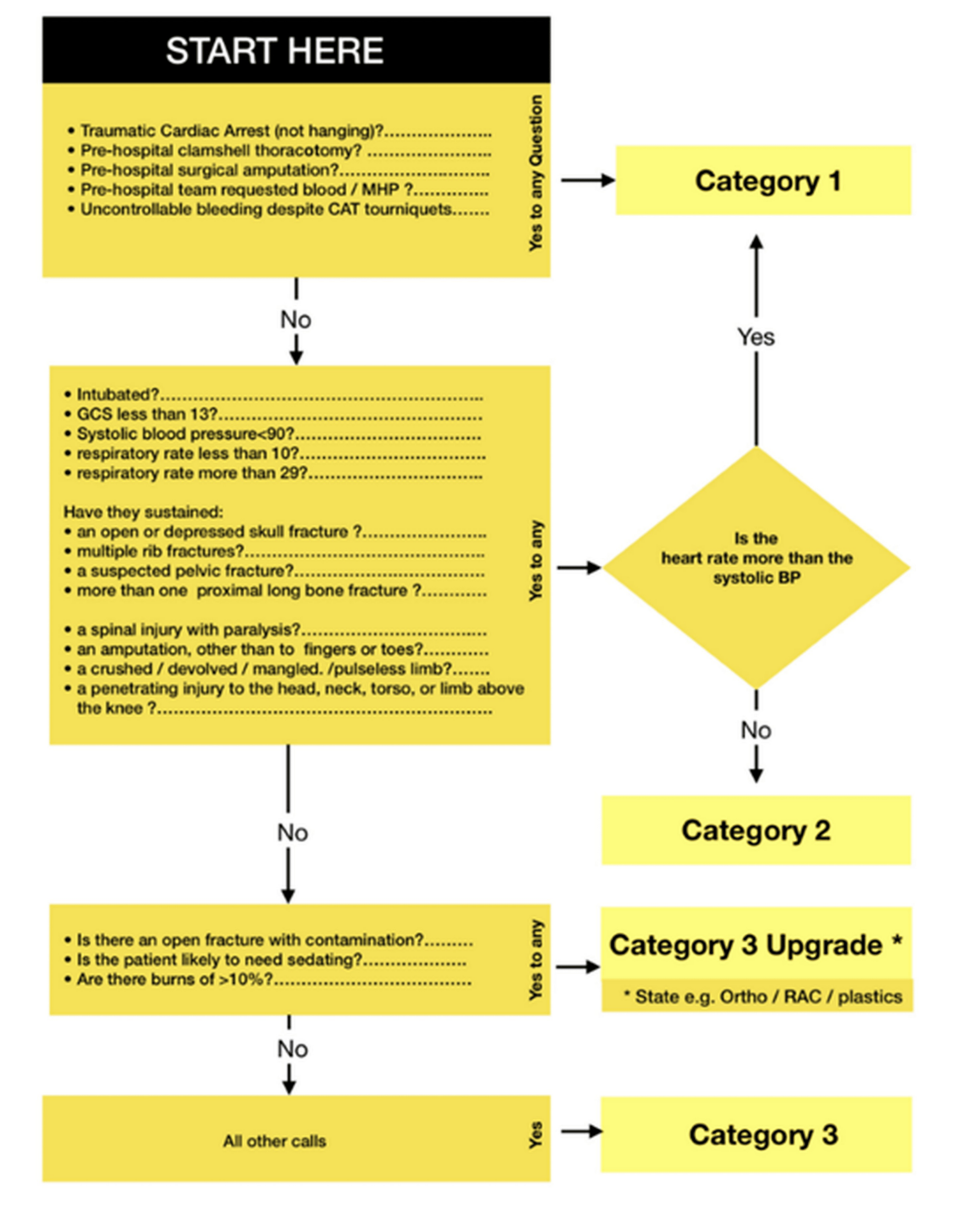
Trauma call categorisation protocol Adapted from the Royal Stoke University Hospital Trauma Team Activation Standard Operating Procedure (June 2019). Reproduced with permission from University Hospitals of North Midlands NHS Trust.

This study is a retrospective audit conducted at Royal Stoke University Hospital (a tertiary major trauma centre), examining Cat1 trauma cases over a seven-month period from October 2024 to April 2025. A total of 37 patients were initially labelled as Cat1 trauma on arrival. The first stage of the audit involved reviewing each patient's initial presentation to determine whether they met the Cat1 criteria (Figure 1) [8]. Further data from this reviewed patient sample were then collected from multiple electronic sources, including prehospital documentation, emergency department trauma notes, imaging reports, and operative records. The key outcome measures included the time from hospital arrival to initial surgical specialty review, whether the appropriate surgical teams were involved based on injury type and severity, and the accuracy of Cat1 labelling. This audit was benchmarked against national guidelines and local trauma protocols where applicable [3,8]. Times were analysed in minutes, with descriptive statistics used to summarise delays and attendance patterns, and comparisons made between expected and actual surgical response times.

## Results

Patient demographics and injury patterns

**Figure 2:**
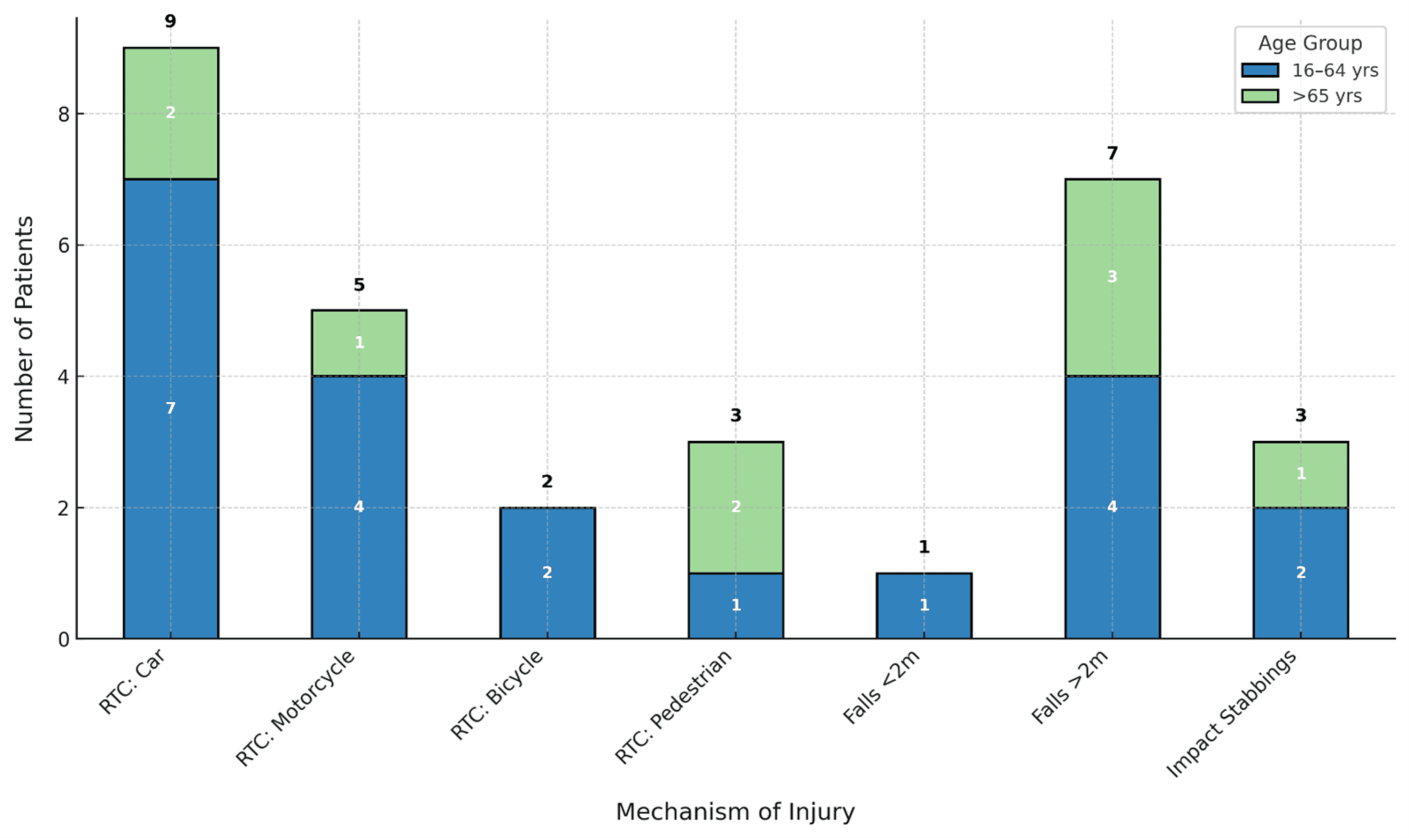
Mechanism of injury by age group (16-64 years and >65 years) A stacked bar graph showing the mechanism of injury of major trauma Cat1 patients presenting to University Hospitals of North Midlands (Royal Stoke University Hospital) between October 2024 and April 2025, stratified by age group (16-64 years and >65 years). RTC: road traffic collision, Cat1: Category 1

On re-review, 30 of the 37 cases labelled as Cat1 trauma were confirmed to meet the criteria. This represents an 81% accuracy rate, with 19% of cases potentially misclassified. While the majority of cases were appropriately labelled, a notable minority appeared to reflect over-triage. Of the 30 confirmed Cat1 trauma cases, 23 patients were male and seven were female. The mean age of the cohort was 49.6 years. A significant proportion of patients sustained injuries from road traffic collisions (RTCs), with car-related RTCs being the most common mechanism, followed by falls from over 2 metres (Figure 2). The majority of the trauma cases occurred in the 16-64 year age group. No injuries were recorded below the age of 16 years.

Mortality and ICU outcomes

The overall mortality rate was high: 20% of patients died within the first 24 hours, and 46.7% died within 30 days or before hospital discharge. Twenty-five (83.3%) patients required ICU admission, and among those who survived longer than 24 hours in the ICU, the mean length of stay was 14.8 days.

Surgical and anaesthetics attendance trends

**Figure 3:**
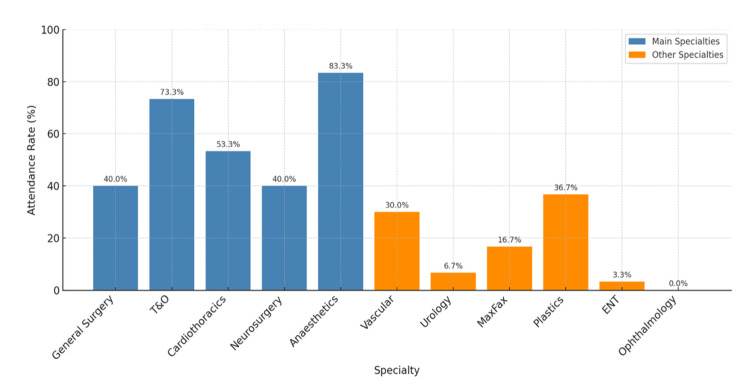
Team attendance in major trauma Cat1 cases (n = 30) A bar graph showing the attendance rates of the core specialities required in Cat1 major trauma cases (blue): general surgery, T&O, cardiothoracics, neurosurgery, and anaesthetics. Attendance rates of other surgical specialities were also collected (orange), including vascular, urology, MaxFax, plastics, ENT, and ophthalmology. T&O: trauma and orthopaedics, ENT: ear, nose, and throat, Cat1: Category 1

Anaesthetics had the highest attendance rate to Cat1 trauma cases at 83.3% (Figure 3). Among the core trauma surgical specialities, T&O attended 73.3% of cases, cardiothoracic 53.3%, and general surgery and neurosurgery each attended 40% of relevant Cat1 trauma cases. Among the non-core specialities, plastic surgery had the highest attendance rate at 36.7%.

Surgical input versus ISS and mortality

**Figure 4:**
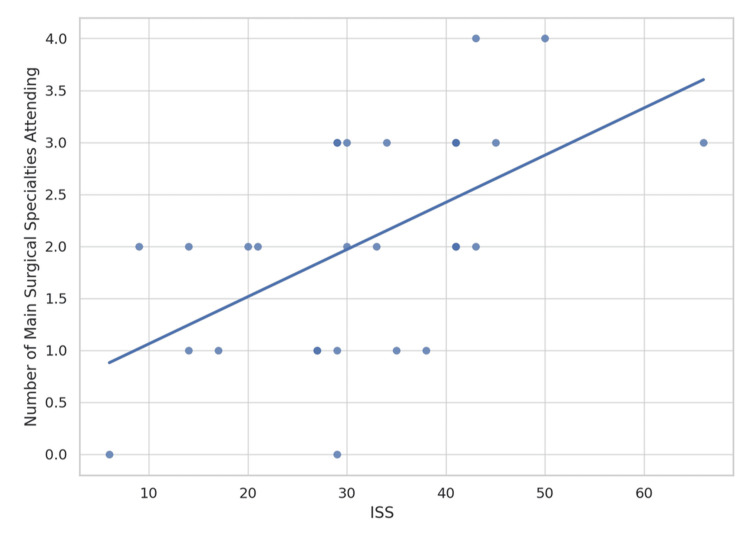
Main surgical specialities attending versus ISS in Cat1 trauma patients Scatter plot of ISS against the number of main surgical specialities attending in the Cat1 trauma cases. ISS: Injury Severity Score, Cat1: Category 1

**Figure 5:**
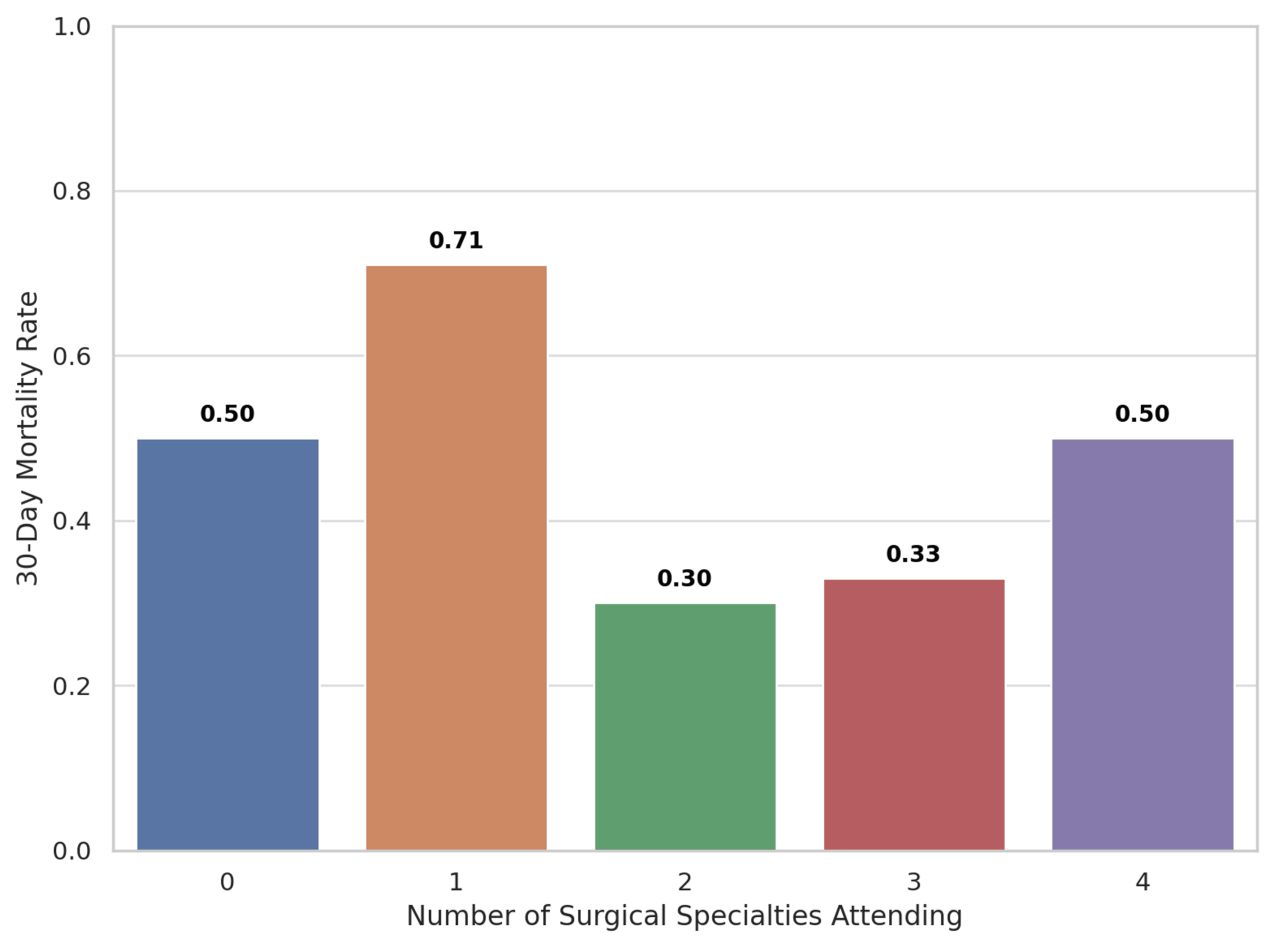
Thirty-day mortality rate by number of surgical specialities attending A bar graph showing the 30-day mortality by the number of core surgical specialities attending in Cat1 trauma cases. Cat1: Category 1

There was a moderate positive correlation between ISS and the number of main surgical specialities attending (r = 0.56, p = 0.001) (Figure 4). This indicates that patients with higher ISS tended to be managed by multiple specialities. The highest 30-day mortality was observed in cases attended by a single core surgical specialty (Figure 5). Mortality was also high when either none or all four core surgical specialities were present. When categorised, patients seen by one or no specialty had a 30-day mortality of 66.7% compared with 33.3% among those reviewed by two or more specialities (Table 1). Although this difference did not reach statistical significance (Fisher's exact test p = 0.12), the effect size was considerable, with patients reviewed by ≤1 specialty being approximately four times more likely to die (odds ratio (OR): 4.0). This trend suggests a potential benefit of broader surgical input, although the limited sample size restricts definitive conclusions.

**Table 1:** Mortality: ≤1 specialty versus ≥2 specialities Comparison of 30-day mortality in Cat1 trauma patients reviewed by ≤1 specialty versus ≥2 specialities. Cat1: Category 1

Group	Deaths	Survivors	Total	Mortality (%)
≤1 specialty	6	3	9	66.70%
≥2 specialties	7	14	21	33.30%
Total	13	17	30	43.30%

Mean time to arrival and damage control surgery to major trauma Cat1

**Figure 6:**
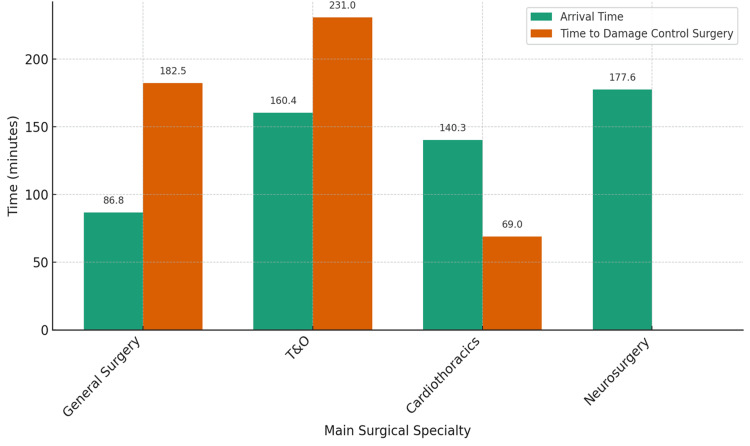
Mean time to arrival and damage control surgery for major trauma Cat1 Comparison of mean arrival time and time to damage control for main surgical specialities in Cat1 major trauma cases. Cat1: Category 1, T&O: trauma and orthopaedics

The mean time from patient arrival in the resuscitation area to surgical team attendance and commencement of damage control surgery, where indicated, varied across the four core surgical specialities involved in Category 1 major trauma cases (Figure 6). General surgery demonstrated the shortest mean arrival time (86.8 minutes), followed by cardiothoracic surgery (140.3 minutes), trauma and orthopaedics (160.4 minutes), and neurosurgery, which showed the longest delay (177.6 minutes). Regarding time to damage control surgery, cardiothoracic surgery achieved the fastest intervention (69 minutes), while trauma and orthopaedics recorded the most prolonged delay (mean: 231 minutes). No damage control procedures were recorded for neurosurgery within the observed dataset. These findings indicate substantial variability in both response and intervention times among the core surgical teams managing major trauma.

Surgical arrival times and outcomes

**Figure 7:**
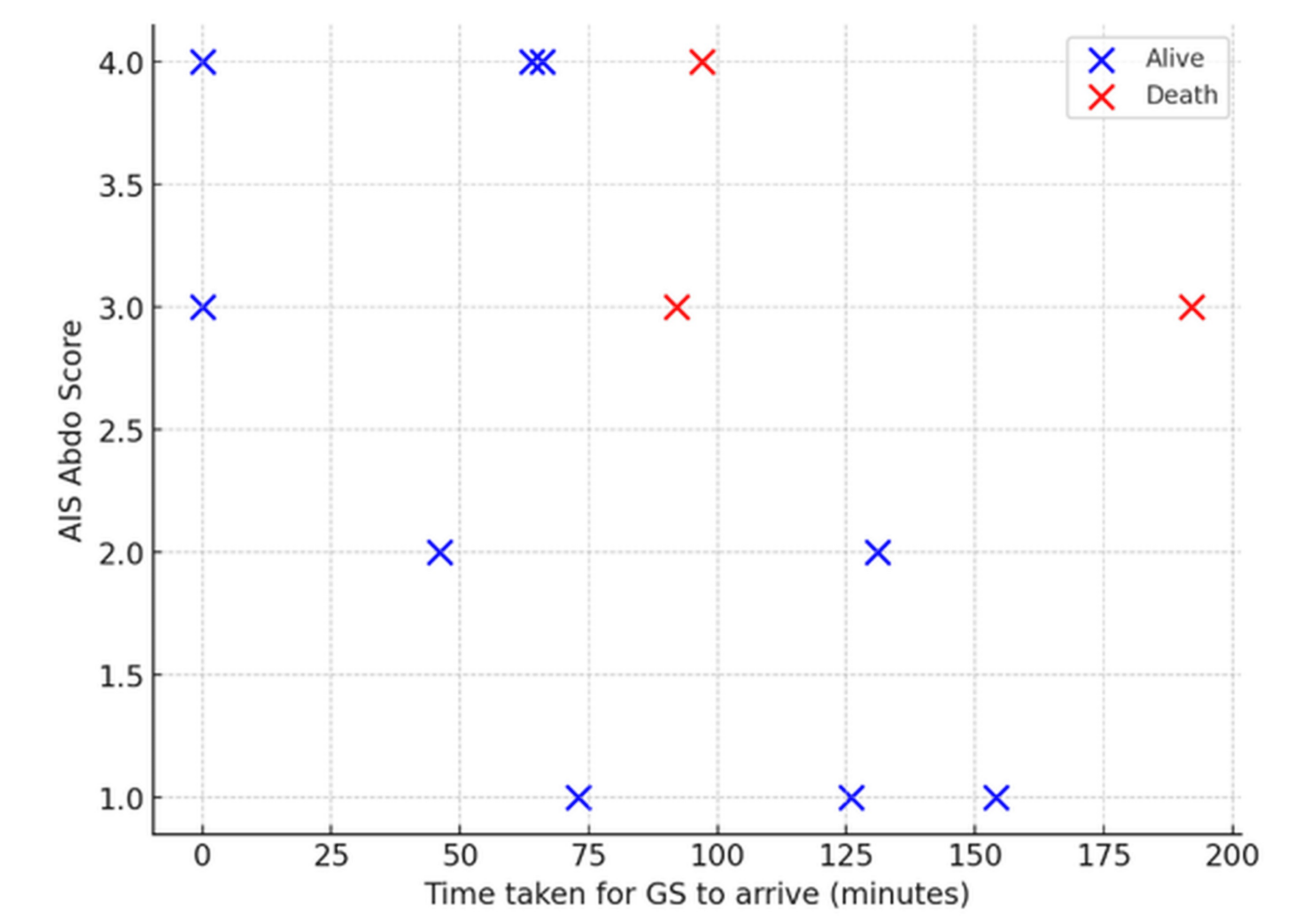
General surgery: time to arrival versus AIS abdominal score Scatter plot showing time to general surgery arrival versus abdominal AIS score in Cat1 trauma patients, further stratified by survival status. AIS: Abbreviated Injury Scale, Cat1: Category 1

In the general surgery cohort (n = 12), time to arrival ranged from 0 to 192 minutes, and AIS abdominal scores ranged from 1 to 4 (Figure 7). Thirty-day mortality occurred in three (25%) patients, all with AIS scores ≥ 3. All patients who died experienced review delays exceeding the 90-minute threshold. No deaths occurred among patients with AIS 1-2 injuries, regardless of time to arrival. Logistic regression incorporating a time × AIS interaction could not be performed due to perfect separation of outcomes by AIS score, indicating that abdominal injury severity was the primary determinant of mortality in this cohort.

**Figure 8:**
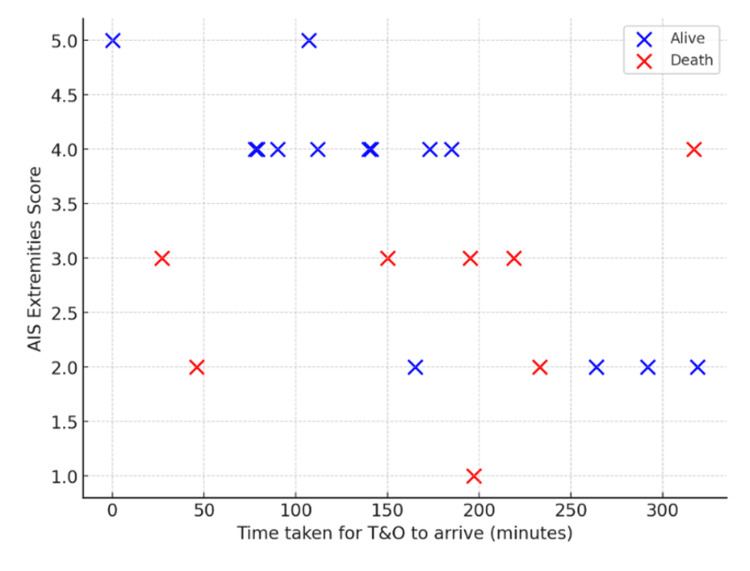
T&O: time to arrival versus AIS extremities score Scatter plot showing time to T&O arrival versus AIS extremities score in Cat1 trauma patients, further stratified by survival status. Cat1: Category 1, T&O: trauma and orthopaedics, AIS: Abbreviated Injury Scale

In the T&O cohort (n = 22), time to arrival ranged from 0 to 319 minutes, and AIS extremities scores ranged from 1 to 5 (Figure 8). Thirty-day mortality occurred in nine (41%) patients. Deaths were most frequent among patients with lower AIS scores (1-3), whereas those with AIS 4-5 injuries largely survived, suggesting an inverse relationship between apparent anatomical severity and outcome.

**Figure 9:**
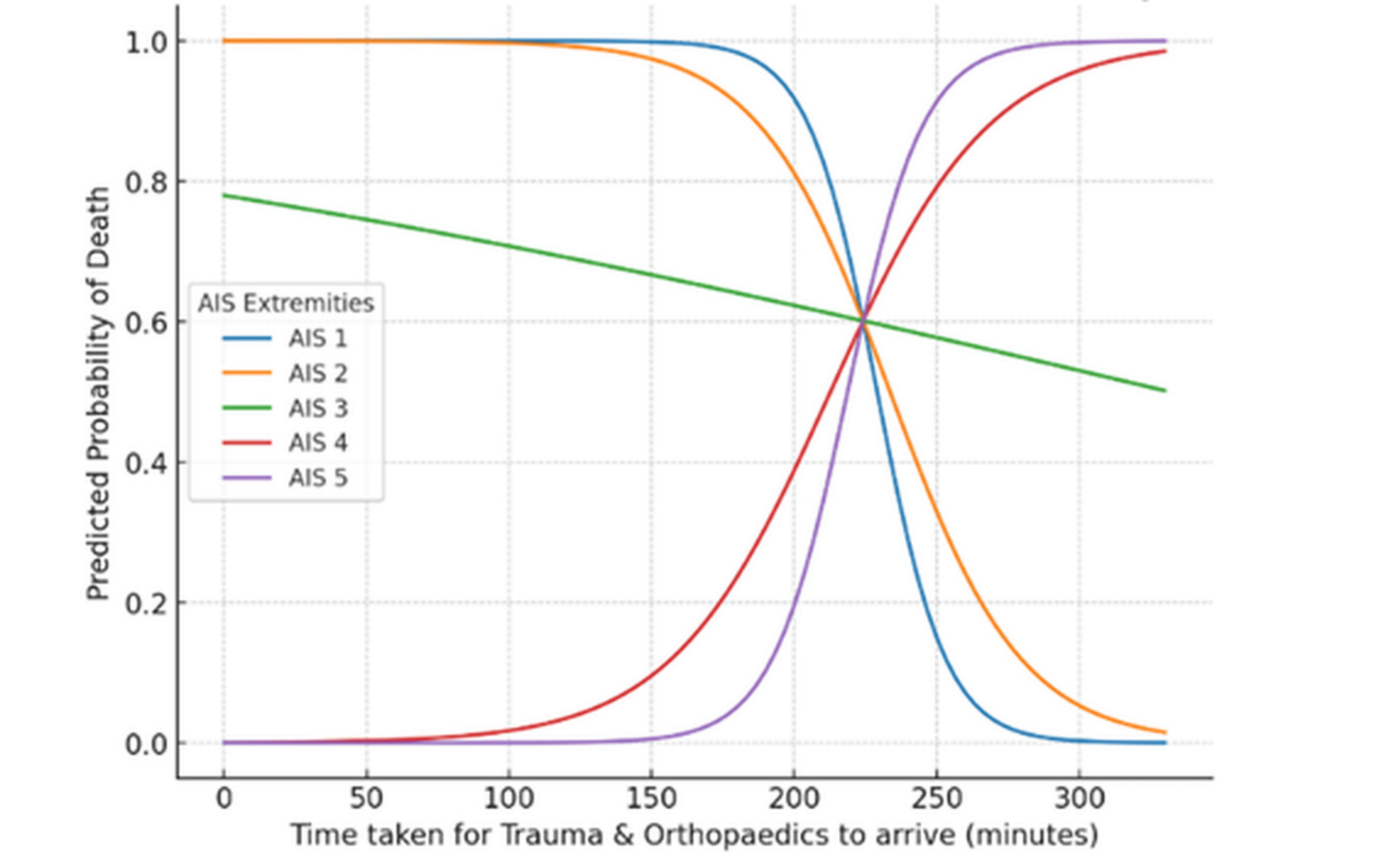
Interaction of AIS extremities and time to arrival on mortality risk (T&Os) Logistic regression model showing predicted probability of death by AIS extremity score and time to T&O arrival. AIS: Abbreviated Injury Scale, T&O: trauma and orthopaedics

Logistic regression with a time × AIS interaction term identified significant main effects but a non-significant interaction (Figure 9). Each additional minute of delay in T&O arrival was associated with lower odds of death (OR: 0.88, 95% confidence interval (CI): 0.79-0.99, p = 0.041), and increasing AIS score was likewise associated with a reduced risk of mortality (OR: 0.00013, 95% CI: 0.000-0.52, p = 0.034). The interaction term approached but did not reach statistical significance (OR: 1.04, 95% CI: 1.00-1.08, p = 0.053). Modelled probability curves demonstrated contrasting patterns across injury severities: for AIS 1-3, predicted mortality decreased with increasing delay, whereas for AIS 4-5, mortality probability rose sharply with prolonged arrival times.

**Figure 10:**
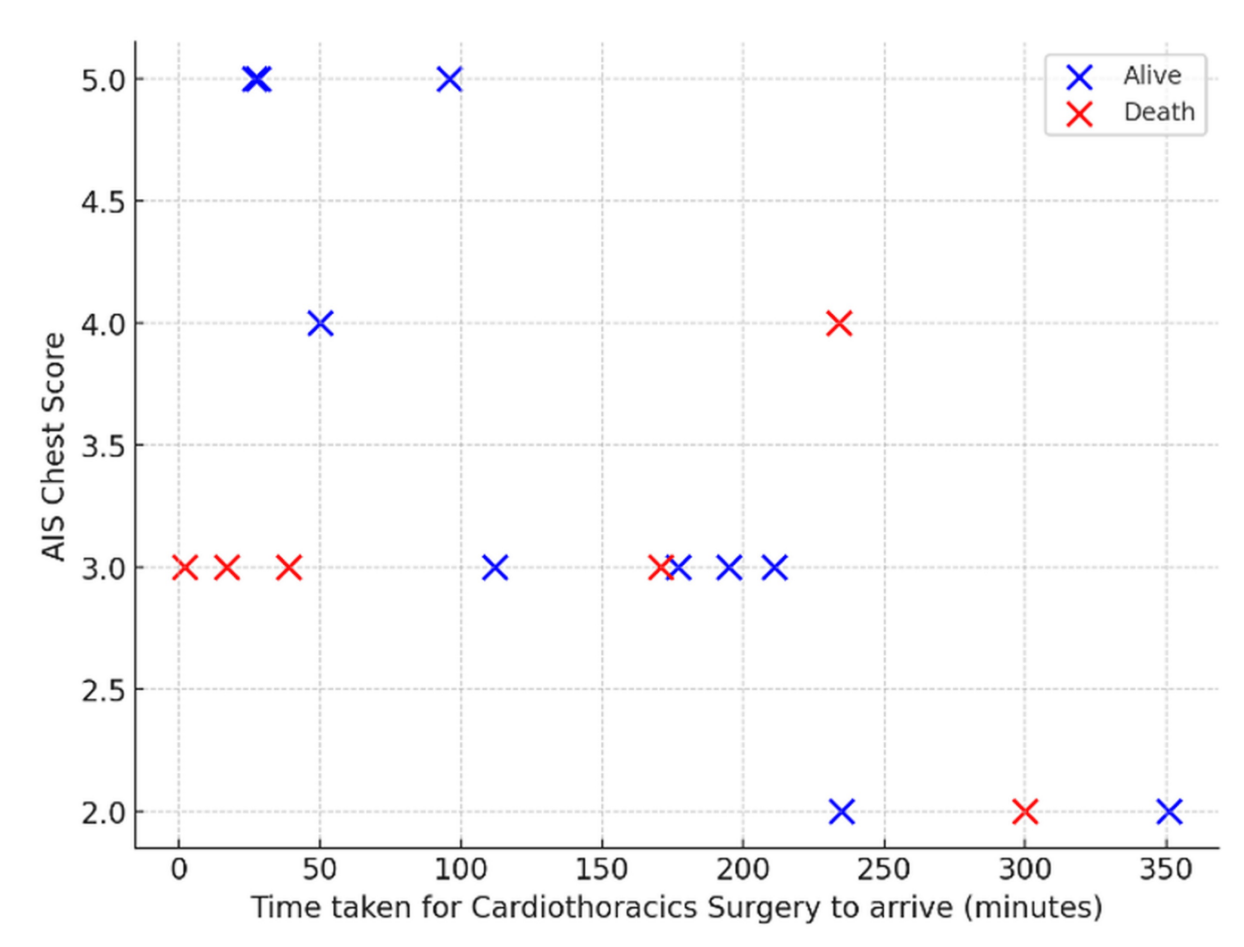
Cardiothoracic surgery: time to arrival versus AIS chest score Scatter plot showing time to cardiothoracic surgery arrival versus AIS chest score in Cat1 trauma patients, further stratified by survival status. AIS: Abbreviated Injury Scale, Cat1: Category 1

In the cardiothoracic surgery cohort (n = 16), time to arrival ranged from 2 to 351 minutes and AIS chest scores from 2 to 5 (Figure 10). Thirty-day mortality occurred in six (38%) patients. Deaths were most frequent among those with AIS scores of 2-3, whereas patients with AIS 4-5 injuries generally survived, suggesting an unexpected inverse relationship between apparent severity and outcome.

**Figure 11:**
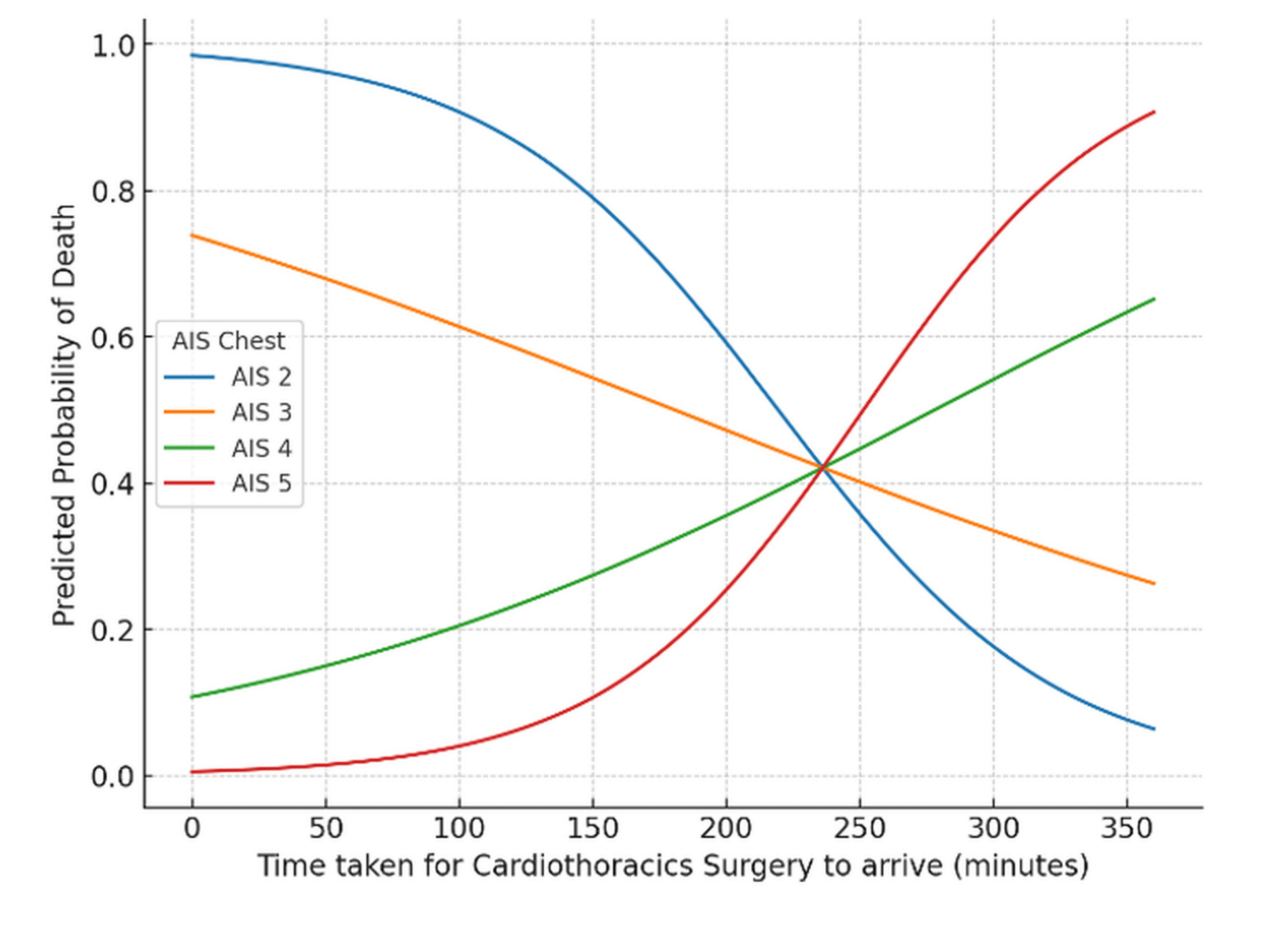
Interaction of AIS chest and time to arrival on mortality risk Logistic regression model showing predicted probability of death by AIS chest score and time to cardiothoracic surgery arrival. AIS: Abbreviated Injury Scale

Logistic regression with a time × AIS interaction term did not demonstrate statistical significance (Figure 11). Each additional minute of delay in cardiothoracic review was associated with a non-significant reduction in the odds of death (OR: 0.96, 95% CI: 0.90-1.02, p = 0.15). Increasing AIS chest score was also not significantly associated with mortality (OR: 0.04, 95% CI: 0.00-3.90, p = 0.17), and the interaction term was non-significant (OR: 1.01, 95% CI: 0.99-1.03, p = 0.21). Modelled probability curves demonstrated distinct patterns across injury severities, with predicted mortality increasing with delay among patients with higher AIS scores (≥4), while remaining low and stable in those with less severe injuries.

**Figure 12:**
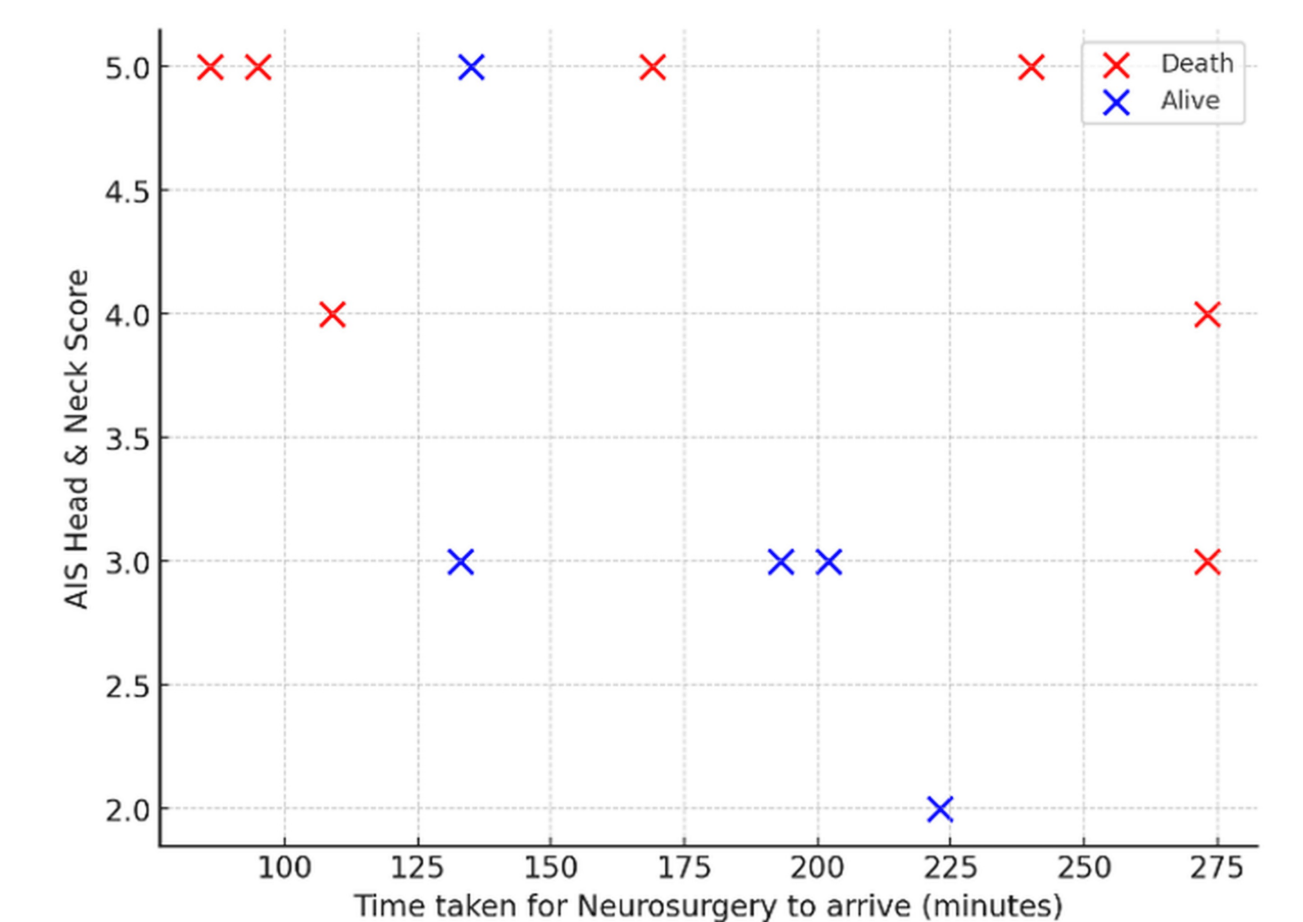
Neurosurgery: time to arrival versus AIS head and neck score Scatter plot showing time to neurosurgery arrival versus AIS head score in Cat1 trauma patients, further stratified by survival status. AIS: Abbreviated Injury Scale, Cat1: Category 1

In the neurosurgery cohort (n = 12), time to arrival ranged from 86 to 273 minutes, and AIS head and neck scores ranged from 2 to 5. Thirty-day mortality occurred in seven (58%) patients, predominantly among those with AIS scores of 4-5 (Figure 12).

**Figure 13:**
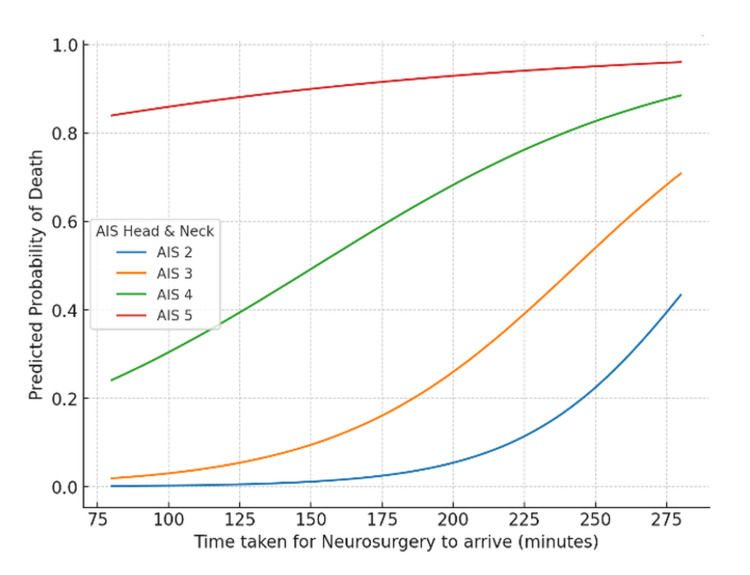
Interaction of AIS head and neck and time to arrival on mortality risk (neurosurgery) Logistic regression model showing predicted probability of death by AIS head score and time to neurosurgery arrival. AIS: Abbreviated Injury Scale

Logistic regression with a time × AIS interaction term did not identify a statistically significant association with mortality (Figure 13). Each additional minute of delay in neurosurgical review was associated with a non-significant increase in the odds of death (OR: 1.05, 95% CI: 0.91-1.21, p = 0.50). AIS head and neck score was also not significantly associated with mortality (OR: 31.97, 95% CI: 0.04-23,487, p = 0.30), and the interaction term was non-significant (OR: 0.99, 95% CI: 0.96-1.03, p = 0.64). Modelled probability curves demonstrated a gradual rise in mortality risk with increasing delay across all AIS categories. Patients with higher AIS scores (4-5) had persistently high baseline mortality that increased further with longer delays, whereas those with lower AIS scores (2-3) started with lower mortality probabilities that rose more steeply with prolonged review times.

## Discussion

This audit evaluated the timeliness of multiple surgical team involvement in Cat1 trauma and explored associations with outcomes. Several key themes emerged, such as variability in surgical response, the influence of injury severity, and the potential importance of broader early specialty involvement. Reassessment showed that Cat1 trauma labelling was accurate in 81% of cases, with 19% of patients classified as Cat1 not meeting the criteria on closer review. This aligns with national experience, where over-triage rates between 15% and 35% are accepted as a trade-off to minimise under-triage and missed critical injuries [9,10]. While over-triage may strain resources, under-triage is associated with increased mortality, justifying a conservative activation threshold [11].

Surgical attendance was inconsistent across specialities, with T&O attending most frequently (73%) and general surgery and neurosurgery less often (40%). This variation reflects findings from other UK trauma centres, where differences in acute care pathways and team activation processes have been noted as factors influencing the timeliness of surgical intervention [12,13]. Early involvement of appropriate surgical teams is integral to the efficiency of a trauma system; delays in consultant review are linked with missed injuries, delayed definitive care, and poorer outcomes [14]. A multi-centre analysis by Haslam et al. demonstrated that time to definitive care within major trauma networks in England remains variable, with delays particularly in cases requiring multiple specialities [4]. Our findings mirror these national patterns, suggesting that current activation processes may not always ensure prompt, coordinated surgical response.

The positive correlation between ISS and the number of surgical specialities attending suggests that mobilisation increases with injury severity (Figure 4). This likely explains the higher mortality observed when all four specialities were present, as these cases generally involved patients with the greatest injury burden rather than reflecting an effect of multidisciplinary attendance itself (Figure 5). Mortality was also substantially higher in patients reviewed by ≤1 specialty compared with ≥2 specialities (66.7% versus 33.3%), suggesting that earlier and broader involvement may enhance decision-making and expedite intervention (Table 1). Although not statistically significant due to small sample size, this four-fold increase in mortality (OR: 4.0) is clinically meaningful. Early multidisciplinary input has been shown to improve trauma outcomes, particularly in complex, multi-system injuries that demand coordinated surgical decision-making [15]. Moreover, studies have demonstrated improved survival when multidisciplinary teams, including surgical specialities, are involved during initial resuscitation [16].

Distinct patterns were observed across specialities. In general surgery, mortality was confined to patients with severe abdominal injuries (AIS ≥3) who also experienced review delays beyond 90 minutes (Figure 7). This suggests that while injury severity was the principal driver of mortality, delayed surgical attendance may have compounded outcomes in these patients. The finding reinforces current National Institute for Health and Care Excellence (NICE) guidance, which emphasises the importance of early surgical review in abdominal trauma and suspected intra-abdominal haemorrhage [3].

For T&O, deaths occurred mainly among patients with lower AIS scores (1-3), likely reflecting physiologically unstable but anatomically less severe injuries such as pelvic fractures (Figure 8). Similar trends are reported in the Trauma Audit and Research Network (TARN) registry, where moderate extremity AIS scores often accompany haemodynamic instability [12]. This pattern highlights a limitation of the AIS scoring system, which may underestimate injury severity in cases where early mortality results from physiological compromise rather than anatomical damage. For more severe injuries in the T&O cohort (AIS 4-5), mortality increased with delayed review, reinforcing the value of early orthopaedic involvement for haemorrhage control and fracture stabilisation (Figure 9) [17].

In the cardiothoracic surgery cohort, mortality was unexpectedly higher among patients with lower AIS scores (Figure 11). Chrysou et al. reported that among severe polytrauma patients, the most common thoracic injuries were rib fractures, minor pneumothoraces, and pulmonary contusions, indicating that relatively minor chest injuries often occur alongside severe multi-region trauma [18]. Similarly, in our cohort, patients with significant extra-thoracic injuries frequently sustained only minor thoracic injuries, resulting in lower thoracic AIS scores despite a substantial overall trauma burden. This highlights the limitation of AIS when interpreted in isolation and reinforces the value of ISS as a more comprehensive measure of injury severity. Although not statistically significant, modelled trends suggested rising mortality with longer review times in patients with higher AIS scores (≥4), implying a possible link between delayed surgical input and poorer outcomes. This is consistent with evidence that prompt thoracic intervention improves survival, as Van Vledder et al. demonstrated that delays to thoracotomy in chest trauma were associated with worse outcomes [19].

In neurosurgery, mortality was highest among patients with severe head injuries (AIS 4-5) and rose further with longer review times, consistent with evidence that early neurosurgical intervention reduces secondary brain injury and mortality after traumatic brain injury (TBI) (Figure 13) [20,21]. Overall, while anatomical severity remained the key determinant of survival, delayed specialist review appeared to exacerbate outcomes across several subspecialities.

These findings support the broader literature on trauma systems and "time to definitive care" as a determinant of survival. Seo et al. demonstrated that in major abdominal trauma, each minute of delay to definitive haemorrhage control was associated with a measurable increase in mortality risk [22]. Similarly, McCullough et al. identified modifiable in-hospital factors such as delayed surgical decision-making and poor inter-specialty communication as key contributors to early deaths in trauma patients [23]. In this context, the delays and variability identified in our audit represent modifiable targets for system improvement.

A structured, automated "surgical bleep system" for Cat1 activations could address these issues. Similar regional trauma system improvements have been shown to enhance coordination and standardise consultant involvement in the early management of major trauma [24]. Such systems align with recommendations from the Royal College of Surgeons (RCS) and NICE NG39, which advocate for immediate senior surgical involvement in major trauma [3,25]. Integrating early activation protocols with prehospital information (mechanism of injury, physiology, and suspected anatomical injuries) could enable anticipatory team assembly even before arrival, reducing in-hospital latency [26].

This audit is limited by its single-centre design, retrospective methodology, and small sample size, which restricted statistical power and increased susceptibility to confounding. AIS-based modelling may under-represent physiological instability, which contributed to outcomes in apparently low-severity groups. Nonetheless, the trends observed are clinically meaningful and provide a foundation for further study. Future work should include prospective or multi-centre evaluation of structured surgical activation systems, ideally linked to TARN for benchmarking against national standards.

## Conclusions

This audit highlights considerable variability in surgical team response times during Cat1 trauma activations, with evidence suggesting that delayed or limited specialist involvement may contribute to poorer outcomes. Although statistical significance was not consistently achieved due to the small sample size, clinically meaningful trends indicate that earlier and broader multidisciplinary input could enhance decision-making, expedite interventions, and potentially improve survival. Implementing a structured "surgical bleep system" for Cat1 trauma calls may help standardise activation processes, ensuring timely engagement of all relevant surgical specialities. Future work should focus on prospective, multicentre evaluation of such systems to optimise trauma response efficiency and outcomes within UK major trauma centres.
